# Restless Legs Syndrome in Dialysis Patients: Does the Dialysis Modality Influence Its Occurrence and Severity?

**DOI:** 10.1155/2018/1414568

**Published:** 2018-02-25

**Authors:** Andreia Freire de Menezes, Douglas Rafanelle Moura de Santana Motta, Fernanda Oliveira de Carvalho, Eduesley Santana-Santos, Manoel Pacheco de Andrade Júnior, Mirela Farias Figueirôa, Maria Isabel Teles Farias, Kleyton de Andrade Bastos

**Affiliations:** ^1^Postgraduate Program in Health Sciences, Federal University of Sergipe, São Cristóvão, SE, Brazil; ^2^Nursing Department, Federal University of Sergipe, São Cristóvão, SE, Brazil; ^3^Clinese Clínica de Nefrologia de Sergipe Ltda., Aracaju, SE, Brazil; ^4^Department of Medicine, Federal University of Sergipe, São Cristóvão, SE, Brazil

## Abstract

**Background:**

Restless legs syndrome (RLS) is more prevalent in chronic kidney patients than in the general population, but it is often diagnosed late and its predictors are unknown.

**Purpose:**

To diagnose RLS in a group of chronic kidney patients on dialysis, determine its frequency and severity, compare the prevalence and severity of the condition among dialytic modalities, and identify possible predictive factors in this population.

**Methods:**

An observational and cross-sectional study with 326 patients who had been on dialysis for more than 3 months, 241 on hemodialysis (HD) and 85 on automatic peritoneal dialysis (APD), using the criteria established by the International Study Group on RLS for the diagnosis and the RLS Rating Scale to determine its severity.

**Results:**

RLS was diagnosed in 19.3% of the patients, 52.4% with severe or very severe forms. Patients with and without RLS did not differ in clinical and demographic characteristics and dialytic modality; however, patients on APD presented higher RLS severity compared to the HD group.

**Conclusions:**

RLS is frequent in dialysis patients and occurs predominantly in its most severe forms; the dialytic modality seems to have no influence on its occurrence; however, it is more severe in patients on APD.

## 1. Introduction

Restless leg syndrome (RLS) is characterized by discomfort, usually in the legs, that causes an overwhelming, irresistible urge to move them, a need that is usually due to unpleasant sensations that worsen during periods of inactivity and that frequently compromises the patient's sleep [1]. It is often diagnosed late, especially when the symptoms are mild or nonspecific [2].

RLS, like other sleep disorders such as insomnia, sleep apnea, and excessive daytime sleepiness, is commonly observed in chronic renal patients, with studies reporting an incidence of 17% to 62% in patients undergoing renal replacement therapy (RRT) [3–8]. The negative impact of these disorders on quality of life (QoL), functional capacity, and survival is well established in the literature [9–15].

The pathophysiology of its occurrence in patients with chronic kidney disease (CKD) is not well established. Authors have proposed several risk factors but the results of the many studies have been inconsistent [6]. The dialytic modality does not seem to interfere in the pathophysiology and incidence of the syndrome [16]; however, recent studies report contrasting results [15, 17].

RLS substantially compromises the quality of life (QoL) of the patient, especially when it occurs in its most severe form and is accompanied by depressive symptoms. Severe forms are usually associated with an increased risk of cardiovascular events and higher mortality [11].

The usual therapeutic measures are generally effective in chronic renal patients, so it is necessary to consider their diagnosis, severity, and identification of possible predictors of their occurrence in this population. And this is what this study aims to accomplish, using a representative sample of patients in a dialysis program.

## 2. Material and Method

This is an observational, cross-sectional study performed with chronic renal patients enrolled in a dialysis program at a reference unit in the city of Aracaju, Sergipe, Brazil. The patients were aged 18 or over, had been on dialysis for at least three months, were clinically stable, were able to communicate verbally, had no mental deficit, and had not undergone kidney transplantation or surgical amputation of the lower limbs. The local ethics committee approved the study (CAAE 00984012.0.0000.0058), and all patients who met the inclusion criteria signed the free and informed form, agreeing to participate in the study.

Data collection took place between April and July 2012. At that time, there were 430 patients on the dialysis program, of whom 326 agreed to take part in the study: 241 on hemodialysis and 85 on automatic peritoneal dialysis. The HD patient interview was performed at the time of dialysis, which occurs on alternate days with duration of 4 hours for each session; the clinic has 3 sessions per day. The APD patients were interviewed on the day of their monthly consultation with the physician. The data collection was carried out by personal interview by a clinical nurse specialist that was trained to administer the questionnaires. Patients were assessed for the presence of RLS by the four criteria of International Restless Syndrome Study Group (IRLSSG) [2] during the face-to-face interview. The questionnaires took approximately 30–50 min to complete. These criteria include unpleasant sensations in foots or legs which develop or are exacerbated during rest; are aggravate in the evening or at night; and are relieve with movement. Patients who fulfilled all four criteria are considered to have RLS and further evaluated by the self-questionnaire of IRLSSG severity scale.

Those who were diagnosed as having RLS answered another questionnaire with ten questions corresponding to the RLS International Rating Scale [18] translated and validated in Portuguese by Masuko et al. [1]. In this questionnaire, all the answers have a score ranging from 0 (corresponding to “none”) to 4 (corresponding to “very large") and the final score represents the sum of the answers of the ten questions, 0 to 10 points, light; 11 to 20 points, moderate; 21 to 30 points, serious; and 31 to 40 points, very serious.

An evaluation form was completed to collect the clinical-demographic information obtained from the electronic medical records of each patient. The following items were included: name, gender, marital status, occupation, schooling, dialysis mode (hemodialysis or peritoneal dialysis), type of vascular access (catheter or arteriovenous fistula), time in dialysis, baseline disease, comorbidities and most recent laboratory exams referring to hemoglobin blood levels, intact parathyroid hormone (iPTH), phosphorus, albumin, and ferritin.

Finally, in order to evaluate the impact of dialysis on the occurrence of RLS and its severity, patients on hemodialysis and peritoneal dialysis were analyzed comparatively.

The information obtained was compared and analyzed using the appropriate statistical methods using the program* Statistical Package for Social Sciences* (SPSS) 16.0 for* Windows* (SPSS Inc., Chicago, Illinois), with *p* < 0.05 being considered for rejection of the null hypothesis.

Descriptive statistics of the studied population were presented in absolute frequency and percentage, in addition to mean and standard deviation and median. In the bivariate statistics, the following tests were applied: Chi-Square and the Odds Ratio (OR) to analyze the relationship between the diagnosis of RLS and categorical independent variables. In the case of ordinal variables, Student's* t-*tests were conducted, maintaining the same dependent variable (RLS diagnosis).

## 3. Results

The 326 patients studied had a mean age of 50.4 ± 15.9 years; 191 patients (58.6%) were men; 194 patients (59.5%) lived with a partner; and 203 patients (77.2%) had been on dialysis for over a year. The majority of the patients underwent hemodialysis (73.9%), through an arteriovenous fistula (59.2%). Hypertensive nephrosclerosis was the most common etiology (26.1%) and in 24.2% of patients the cause of CKD was not identified. Systemic arterial hypertension was the main comorbidity identified (271 patients, 83.1%). Sixty-three patients (19.3%) were diagnosed as having RLS.

Tables 1 and 2 show the distribution of patients according to the dialytic modality and clinical-demographic characteristics, segregated according to the diagnosis of RLS. Patients with and without RLS did not differ in the variables studied.

The 63 patients with RLS answered the 10 questions of the International Rating Scale for the disease (1): 7.9% presented mild discomfort, 39.7% moderate discomfort, 32.8% severe discomfort, and 19.6% very severe discomfort. 67.3% of patients reported that their need to move their limbs was moderate to great; however, approximately 45% reported complete relief of discomfort when walking. The majority of patients reported that RLS was associated with impaired sleep quality (67.2%) and fatigue or somnolence (63.8%), of varying degrees of intensity. The symptoms in 39.7% of the cases lasted less than one hour per day; however 43.1% of the individuals reported that they occurred almost daily. For 37.9% of the interviewees, the symptoms of RLS did not affect their ability to perform normal activities, but 72.4% reported mood changes of varying degrees of intensity.

**Figure 1:**
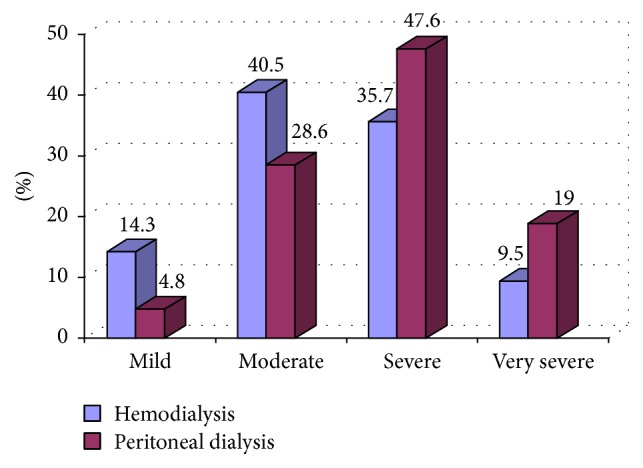
Percentage distribution of patients with restless legs syndrome in terms of their severity, according to the dialysis modality (*N* = 63).

According to the results of the RLS International Rating Scale, most patients (52.4%) had RLS in its severe or very severe forms (Figure 1).

RLS was diagnosed in 17.4% of the individuals who underwent hemodialysis, and in those patients the disease was mild or moderate (54.8%). With regard to patients who underwent peritoneal dialysis, RLS was identified in 24.7% of them and there was greater penetration of severe or very serious disease (66.6), but no statistically significant differences were observed between the two groups in terms of prevalence (*p* = 0.11).

## 4. Discussion

In this study we diagnosed RLS in 19.3% of the chronic renal patients who were in a dialysis program, and in 52.4% of them the disease was characterized as severe or very serious. No independent predictor of this syndrome was identified in the study population. Additionally, it was verified that patients with RLS on peritoneal dialysis presented different profiles in terms of the prevalence and severity of the disease compared to those on hemodialysis but with no statistical significance.

It is estimated that RLS has a prevalence in the general population varying from 2 to 15%, depending on the characteristics of the individuals and the diagnostic criteria used, and that it is a common disorder in chronic renal patients in renal replacement therapy RRT [19]. Studies report rates of diagnosis of the disease in this population ranging from 17 to 62% [3–7]. The pathophysiology of RLS is still obscure, and its genesis may be uremia, as well as iron deficiency. It is thought to be a peripheral disorder, but studies of dopamine metabolism in the brain raise the possibility of central nervous system (CNS) origin, more specifically by the organic deficiency of hypothalamic dopaminergic cells that are the source of dopamine for the spinal cord [20]. Iron acts as a cofactor for the enzyme tyrosine hydroxylase, an important step in the synthesis of dopamine in the CNS. Thus, low levels of serum iron would cause a decrease in dopamine production, which, in itself, could lead to RLS [21].

As reported for the general population, a strong association between RLS and serum levels of iron and ferritin has been reported in chronic renal failure, which is generally below 40 ng/mL [3, 13, 22–24]; however, more recent studies have not confirmed these findings [21, 25]. Additionally, in dialysis patients it has been suggested that anemia, regardless of iron stores, may be the major cause of RLS development [14]. Patients with altered renal function produce less erythropoietin, with a consequent reduction of erythropoiesis, which, in a not yet fully understood way, reduces the cotransport of iron to the CNS and medulla [21].

Regarding hemoglobin and hematocrit levels, the results diverge, in a study with hemodialysis patients only; there was a relationship between RLS in dialysis patients and a decrease in hemoglobin (*p* < 0.005) [26]; however, other recent studies have not found any type of association [13, 17]. In a recent meta-analysis by Mao et al. [27], in which 23 studies were included, dialysis patients with RLS had markedly lower levels of hemoglobin (Hb)/iron compared to non-RLS in global populations.

The median values of the laboratory test results for hemoglobin, iPTH, albumin, and phosphorus were in agreement with that recommended for this population. The median ferritin was above the recommended level. However, in 59.2% of the patients, the values were within the normal range, with only 14 patients (4.3%) having ferritin levels < 100 ng/mL, two of whom were diagnosed as having RLS (3.2% of RLS cases) [28]. However, it should be borne in mind that this marker reflects iron stores more accurately when they are reduced, since inflammation is highly prevalent in this population, and liver disease contributes to its elevation [29]. In addition, dialysis patients generally have strict hematimetric and iron store control, with the almost continuous use of erythropoiesis and iron supplementation agents.

CKD combined with hemodialysis, the most common dialysis modality, are among the chronic pathologies and therapies that most affect patients' QoL, with depression being a relatively common psychiatric disorder in this population. More broadly, a complex interaction between depression, QoL, clinical complications, and survival is observed in dialysis patients [30]. It has been shown that RLS can add to this effect and further substantially compromise the QoL of those it affects, with the most important factors being its severity and the presence of depressive symptoms [31]. Tuncel et al. [13] reported that the presence of RLS in hemodialysis patients negatively affects QoL and contributes to the occurrence of depression. In a cross-sectional study, Szentkiralyi et al. [12] found that those with chronic kidney disease and RLS had a higher prevalence of depressive symptoms than those without RLS (56% versus 22%, *p* < 0.001).

In this series, we did not study the associations between RLS, QoL, and depression; however, in our previous study, in the same place, Salman [32] reported a significant lowering of the QoL level among 114 chronic hemodialysis patients, mainly in regard to physical aspects. Depression, with a prevalence of 28.9%, was the highest predictor of QoL, being associated with lower scores in all dimensions of the evaluation instrument used. A similar result was found in a recent study. Of the 400 patients interviewed, 19.3% had depressive symptoms and the main independent factors were poor sleep quality, unemployment, diabetes, hypoalbuminemia, low education, and pruritus [26].

In the present analysis, we observed that the majority of patients had severe or very severe RLS, and it was notable that 67.2% of them reported impaired sleep quality, 63.8% reported drowsiness, 62.1% reported that the symptoms affected the performance of routine activities, and 72.4% reported having mood changes. These results corroborate current data showing that RLS is associated with poor sleep quality, excessive daytime sleepiness, depressive symptoms, and increased risk of obstructive sleep apnea [26].

Increased severity of RLS has been associated with an increased risk of cardiovascular events and increased mortality and is thought to play a role in the pathogenesis of hypertension during sleep [11, 14]. Considering what has been reported in the literature, it is plausible that, due to the intensity of the symptoms observed in our patients, there is a substantial impairment in their QoL [13, 31] and a higher cardiovascular risk [11, 14].

Considering that in the bivariate analysis there was no evidence of an association between the characteristics, a multivariate analysis to identify possible independent predictors for RLS was not performed, since no statistical significance was found in the bivariate analysis between the characteristics considered and restless leg syndrome. When segregating patients according to the dialysis modality, although there is no statistical significance in terms of numbers, the severity profile of the disease appears to be different. There was a higher prevalence of RLS (24.7 versus 17.4%) and a higher percentage of patients perceived to be more severe (66.7 versus 45.2%) in patients on peritoneal dialysis than in those on hemodialysis. The quantitative study of individuals on peritoneal dialysis (85 patients) submitted to RLS evaluation in this study contrasts with the majority of publications in which only hemodialysis patients are considered in the analysis, perhaps because this is the predominant modality in most countries [32, 33]. Recent studies report contrasting results: Al-Jahdali [17] reported a significantly higher prevalence of RLS in APD patients than in hemodialysis patients (69 versus 46%). However, Merlino et al. [15], investigating 86 patients (67.4% on hemodialysis), identified a higher prevalence of RLS in hemodialysis patients (19%) than in patients on APD (10.7%).

## 5. Conclusions

RLS is a common disease in dialysis patients and occurs predominantly in its most severe forms and should be investigated early and routinely in these patients, in order to apply the known effective therapeutic measures and prevent its well described negative outcomes. The dialytic modality does not seem to influence the occurrence of RLS and we did not identify other factors independently associated with it in this population. Further studies are needed to confirm whether other clinical-demographic factors predict its onset.

It is worth noting that this study presents a high number of patients compared to the articles already published; however, it presents the limitation of not being multicentric, and the lack of association between the characteristics considered and RLS did not allow us to identify independent predictors through a multivariate analysis.

Care of a dialysis patient should include special attention to the diagnosis and treatment of RLS, as it has a high prevalence in relation to the general population and presents in a severe form in this population, particularly in those on APD. Additional studies are needed in an attempt to identify possible predictors, since the characteristics analyzed in this study were not associated with the diagnosis.

## Figures and Tables

**Table 1:** Distribution of hemodialysis patients according to clinical-demographic characteristics, segregated according to the diagnosis of restless legs syndrome (RLS)^¢^ (*N* = 241).

Characteristics	Population	With RLS	Without RLS	*p* ^∂^
	100% (241)	17.4% (42)	82.6 (199)	-
Age (average), years	48.9 ± 15.7	48.1 ± 13.7	49.1 ± 16.1	0.71
Male, %	58.5	50.0	60.3	0.22
Arterial hypertension, %	83.1	78.6	84.0	0.40
Diabetes mellitus, %	26.6	19.0	28.1	0.22
Cardiopathy, %	23.2	30.9	21.6	0.19
Peripheral vasculopathy, %	14.9	19.0	14.1	0.41
Having a partner, %	56.4	69.0	53.8	0.07
Years of study, <4, %	51.0	52.4	50.7	0.84
Regular occupation, %	8.3	9.5	8.0	0.75
Time on dialysis, >1 year, %	80.1	80.9	79.9	0.87
Hemoglobin (g/dL)^*ø*^	10.8	10.7	10.9	0.70
Parathormone (pg/mL)^*ø*^	580.7	621.6	496.1	0.78
Phosphorus (mq/dL)^*ø*^	4.8	4.9	4.7	0.13
Albumin (g/dL)^*ø*^	3.8	3.8	3.8	0.54
Ferritin (ng/mL)^*ø*^	670.3	671.3	625.5	0.94

^¢^Based on the criteria developed by the International Study Group on Restless Legs Syndrome (18). ^*ø*^Median of values obtained from blood samples. Significance calculated from mean values. ^∂^Significance level *p* < 0.05.

**Table 2:** Distribution of peritoneal dialysis patients according to clinical-demographic characteristics, segregated according to the diagnosis of restless legs syndrome (RLS)^¢^ (*N* = 85).

Characteristics	Population	With RLS	Without RLS	*p* ^∂^
	100% (85)	24.7% (21)	75.3% (64)	-
Age (average), years	56.5 ± 15.3	59.3 ± 13.8	53.0 ± 17.5	0.14
Male, %	57.1	71.4	54.7	0.18
Arterial hypertension, %	83.9	90.5	81.2	0.50
Diabetes mellitus, %	32.1	38.0	32.8	0.66
Cardiopathy, %	28.6	23.8	34.4	0.37
Peripheral vasculopathy, %	16.1	19.0	18.7	1.00
Having a partner, %	73.2	61.9	70.3	0.47
Years of study, <4, %	58.9	47.6	65.6	0.14
Regular occupation, %	16.1	19.0	15.6	0.74
Time on dialysis, >1 year, %	67.9	61.9	68.7	0.56
Hemoglobin (g/dL)^*ø*^	11.0	11.9	11.1	0.60
Parathormone (pg/mL)^*ø*^	457.7	355.5	396.0	0.41
Phosphorus (mq/dL)^*ø*^	4.9	3.9	4.84	0.33
Albumin (g/dL)^*ø*^	3.5	3.4	3.4	0.40
Ferritin (ng/mL)^*ø*^	704.3	723.5	746.8	0.59

^¢^Based on the criteria developed by the International Study Group on Restless Legs Syndrome (18). ^*ø*^Median values. ^∂^Significance level, *p* < 0.05.

## References

[1] Masuko A. H., Carvalho L. B. C., Machado M. A. C., Morais J. F., Prado L. B. F., Prado G. F. (2008). Translation and validation into the Brazilian Portuguese of the restless legs syndrome rating scale of the International Restless Legs Syndrome Study Group. *Arquivos de Neuro-Psiquiatria*.

[2] Allen R. P., Picchietti D., Hening W. A., Trenkwalder C., Walters A. S., Montplaisi J. (2003). Restless legs syndrome: diagnostic criteria, special considerations, and epidemiology. A report from the restless legs syndrome diagnosis and epidemiology workshop at the National Institutes of Health. *Sleep Medicine*.

[3] Hui D. S. C., Wong T. Y. H., Ko F. W. S. (2000). Prevalence of sleep disturbances in Chinese patients with end-stage renal failure on continuous ambulatory peritoneal dialysis. *American Journal of Kidney Diseases*.

[4] Thorp M. L. (2001). Restless legs syndrome. *International Journal of Artificial Organs*.

[5] Mucsi I., Molnar M. Z., Ambrus C. (2005). Restless legs syndrome, insomnia and quality of life in patients on maintenance dialysis. *Nephrology Dialysis Transplantation *.

[6] Kavanagh D., Siddiqui S., Geddes C. C. (2004). Restless Legs Syndrome in Patients on Dialysis. *American Journal of Kidney Diseases*.

[7] Kawauchi A., Inoue Y., Hashimoto T. (2006). Restless legs syndrome in hemodialysis patients: Health-related quality of life and laboratory data analysis. *Clinical Nephrology*.

[8] Bastos M. G. (2011). Kirsztajn GM: Chronic kidney disease: importance of early diagnosis, immediate referral and structured interdisciplinary approach to improve outcomes in patients not yet on dialysis. *Jornal Brasileiro de Nefrologia*.

[9] Benz R. L., Pressman M. R., Hovick E. T., Peterson D. D. (2000). Potential novel predictors of mortality in end-stage renal disease patients with sleep disorders. *American Journal of Kidney Diseases*.

[10] Happe S., Klösch G., Saletu B., Zeitlhofer J. (2001). Treatment of idiopathic restless legs syndrome (RLS) with gabapentin. *Neurology*.

[11] Portaluppi F., Cortelli P., Buonaura G. C., Smolensky M. H., Fabbian F. (2009). Do restless legs syndrome (RLS) and periodic limb movements of sleep (PLMS) play a role in nocturnal hypertension and increased cardiovascular risk of renally impaired patients?. *Chronobiology International*.

[12] Szentkiralyi A., Molnar M. Z., Czira M. E. (2009). Association between restless legs syndrome and depression in patients with chronic kidney disease. *Journal of Psychosomatic Research*.

[13] Tuncel D., Orhan F. Ö., Sayarlioglu H., IsIk I. O., Utku U., Dinc A. (2011). Restless legs syndrome in hemodialysis patients: Association with depression and quality of life. *Sleep and Breathing*.

[14] La Manna G., Pizza F., Persici E. (2011). Restless legs syndrome enhances cardiovascular risk and mortality in patients with end-stage kidney disease undergoing long-term haemodialysis treatment. *Nephrology Dialysis Transplantation *.

[15] Merlino G., Lorenzut S., Romano G. (2012). Restless legs syndrome in dialysis patients: A comparison between hemodialysis and continuous ambulatory peritoneal dialysis. *Neurological Sciences*.

[16] Janzen L., Rich J. A., Vercaigne L. M. (1999). An overview of levodopa in the management of restless legs syndrome in a dialysis population: Pharmacokinetics, clinical trials, and complications of therapy. *Annals of Pharmacotherapy*.

[17] Al-Jahdali H. (2011). A comparison of sleep disturbances and sleep apnea in patients on hemodialysis and chronic peritoneal dialysis. *Saudi Journal of Kidney Diseases and Transplantation*.

[18] Adabag A. S., Ishani A., Bloomfield H. E., Ngo A. K., Wilt T. J. (2009). Efficacy of N-acetylcysteine in preventing renal injury after heart surgery: a systematic review of randomized trials. *European Heart Journal*.

[19] Allen R. P., Bharmal M., Calloway M. (2011). Prevalence and disease burden of primary restless legs syndrome: Results of a general population survey in the United States. *Movement Disorders*.

[20] Clemens S., Rye D., Hochman S. (2006). Restless legs syndrome: revisiting the dopamine hypothesis from the spinal cord perspective. *Neurology*.

[21] Goffredo Filho G. S., Gorini C. C., Purysko A. S., Silva H. C., Elias I. E. F. (2003). Restless legs syndrome in patients on chronic hemodialysis in a Brazilian city: Frequency, biochemical findings and comorbidities. *Arquivos de Neuro-Psiquiatria*.

[22] Earley C. J., Connor J. R., Beard J. L., Malecki E. A., Epstein D. K., Allen R. P. (2000). Abnormalities in CSF concentrations of ferritin and transferrin in restless legs syndrome. *Neurology*.

[23] Allen R. P., Barker P. B., Wehrl F., Song H. K., Earley C. J. (2001). MRI measurement of brain iron in patients with restless legs syndrome. *Neurology*.

[24] Silber M. H., Richardson J. W. (2003). Multiple blood donations associated with iron deficiency in patients with restless legs syndrome. *Mayo Clinic Proceedings*.

[25] Kim J.-M., Kwon H.-M., Lim C. S., Kim Y. S., Lee S.-J., Nam H. (2008). Restless legs syndrome in patients on hemodialysis: Symptom severity and risk factors. *Journal of Clinical Neurology*.

[26] Araujo S. M. H. A., Bruin V. M. S. D., Nepomuceno L. A. (2010). Restless legs syndrome in end-stage renal disease: Clinical characteristics and associated comorbidities. *Sleep Medicine*.

[27] Mao S., Shen H., Huang S., Zhang A. (2014). Restless legs syndrome in dialysis patients: A meta-analysis. *Sleep Medicine*.

[28] (2006). KDOQI, Foundation NK: KDOQI Clinical Practice Guidelines and Clinical Practice Recommendations for Anemia in Chronic Kidney Disease. *American Journal of Kidney Diseases*.

[29] Kowdley K. V., Belt P., Wilson L. A. (2012). Serum ferritin is an independent predictor of histologic severity and advanced fibrosis in patients with nonalcoholic fatty liver disease. *Hepatology*.

[30] Kimmel P. L. (2001). Psychosocial factors in dialysis patients. *Kidney International*.

[31] Happe S., Reese J. P., Stiasny-Kolster K. (2009). Assessing health-related quality of life in patients with restless legs syndrome. *Sleep Medicine*.

[32] Salman S. M. (2011). Restless legs syndrome in patients on hemodialysis. *American Journal of Kidney Diseases*.

[33] Malaki M., Mortazavi F. S., Moazemi S., Shoaran M. (2012). Insomnia and limb pain in hemodialysis patients: what is the share of restless leg syndrome?. *Saudi Journal of Kidney Diseases and Transplantation*.

